# The Insertion of Threaded Acetabulum Components: Indications According to Specific Acetabulum Anatomy and Methods to Avoid Pitfalls

**DOI:** 10.7759/cureus.50824

**Published:** 2023-12-20

**Authors:** Christos Koutserimpas, Ilias Karaiskos, Maria Piagkou

**Affiliations:** 1 Department of Orthopaedics and Traumatology, "251" Hellenic Air Force General Hospital, Athens, GRC; 2 Anatomy/Oral Surgery, National and Kapodistrian University of Athens, Athens, GRC

**Keywords:** acetabulum anatomical characteristics, hip prostheses, acetabulum, hip anatomy, total hip arthroplasty, hip surgery

## Abstract

New-generation threaded acetabulum components have been used in total hip arthroplasty (THA) with good outcomes. We have extensively used the EcoFit® SC cup (Implantcast, Buxtehude, Germany) in our practice. In this report, we present some major complications related to the use of this implant, as well as insights regarding the surgical technique to avoid such adverse effects. Furthermore, we attempt to describe certain contraindications for using threaded cups in THA, taking into account specific patient anatomy and intraoperative acetabulum preparation. We have observed acetabulum roof and posterior wall fractures, as well as incomplete placement of the component. Ensuring the meticulous preparation of the peripheral rim of the acetabulum is crucial to prevent incomplete placement since threaded components have a larger diameter than that of the reamers used to prepare the acetabulum. Additionally, when dealing with the acetabula where the posterior or anterior walls have thinned, it is advisable to refrain from using a threaded cup to avoid the risk of intraoperative fractures caused by the torque forces exerted during implant insertion.

## Introduction

In the 1970s, cementless acetabular cups were introduced as a solution to combat the "cement disease" [1]. This condition involves the gradual loss of bone tissue due to tiny particles accumulating at the junction between cement and bone. It is considered the primary reason for the failure of cemented polyethylene cups due to aseptic loosening [2]. Over the past two decades, long-term data have emerged to endorse acetabular components as the preferred method. Della Valle et al. documented that cementless acetabular components offer robust fixation, with a remarkable 96% survival rate at the 20-year mark after surgery [3]. Apart from cement, the primary fixation of acetabular components can be attained through a variety of methods, including press-fit or threaded approaches, with or without the use of screws [1,3].

New-generation threaded acetabular cups have demonstrated excellent initial stability, a crucial factor for facilitating successful osteointegration and thereby ensuring long-term secondary stability [4]. The primary stability of threaded components is achieved through a press-fit mechanism, where a threaded portion is securely anchored within the bone [4,5]. This primary stability relies on various factors, including the cup's rim design, geometry, implant material, and surface finish, as well as the variously designed teeth and the cup's own structural configuration [4,5]. Threaded cups can be classified into two primary shapes: conical and spherical, along with a range of hybrid variations that exhibit similar functional characteristics [4-7].

The threads can incorporate sharp, serrated, or flat teeth, as well as various sub-patterns and hybrid forms, each varying in terms of the number, size, surface area, curvature, thread pitch, and chip flute [4-7]. These parameters significantly impact the cup's insertion process and its initial stability. In particular, sharp teeth enable more aggressive penetration into the bone, providing excellent initial fixation, while flat teeth might provide a more evenly distributed stress load, minimizing the risk of stress concentration. Furthermore, the number of teeth, their size, and curvature influence how the cup engages with the bone. A larger surface area increases bone-to-implant contact, promoting osseointegration (the fusion of the bone with the implant), while curvature is designed to match the natural contours of the acetabulum for better fit and stability [4,5]. Spherical cups mimic the natural shape of the acetabulum, providing good stability and range of motion, while conical ones offer inherent stability due to their tapering design, enabling secure fixation within the bone [4-7].

We have extensively used, in our practice, the EcoFit® SC cup (Implantcast, Buxtehude, Germany) with good outcomes, implant survival, and subjective and objective patient satisfaction rates, as already reported in the literature [8]. Nevertheless, we have experienced a few major complications as well, such as acetabulum fractures, and the superficial placement of the cup (incomplete placement). In this technical note, we provide typical examples of these major complications and thoroughly describe the insertion technique of this acetabulum component, so that these adverse effects can be avoided. Furthermore, we attempt to define the contraindications for the threaded acetabulum components in total hip arthroplasty (THA).

## Technical report

Examples of major complications

**Figure 1:**
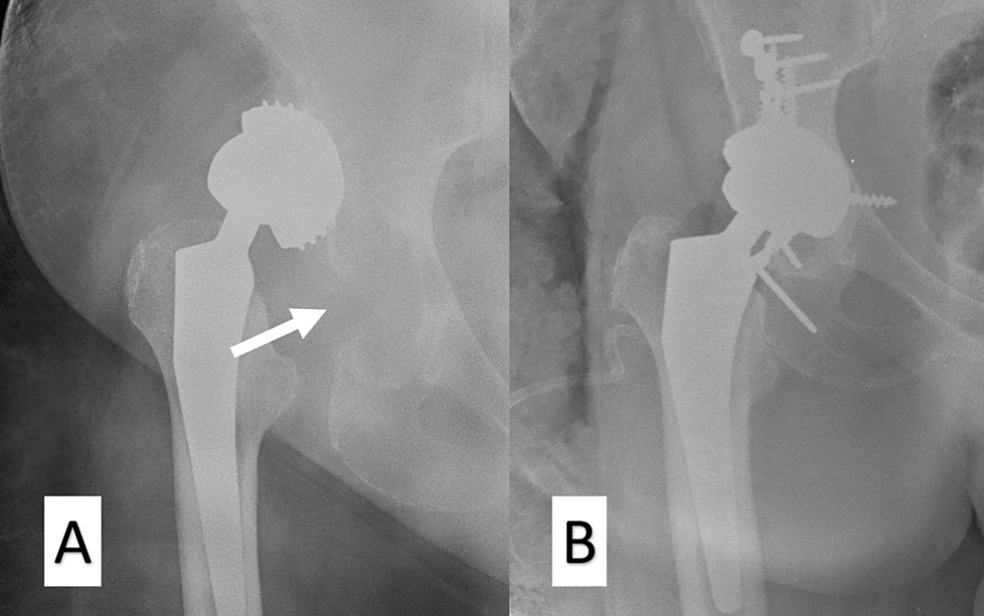
Postoperative anteroposterior X-rays of the patient A: Postoperative anteroposterior X-ray view after mobilization of the patient. A posterior wall acetabulum fracture was noted (white arrow), while the threaded cup had migrated proximally. B: Anteroposterior X-ray view after revision surgery. Open reduction and internal fixation and placement of a press-fit multi-hole cup were done

**Figure 2:**
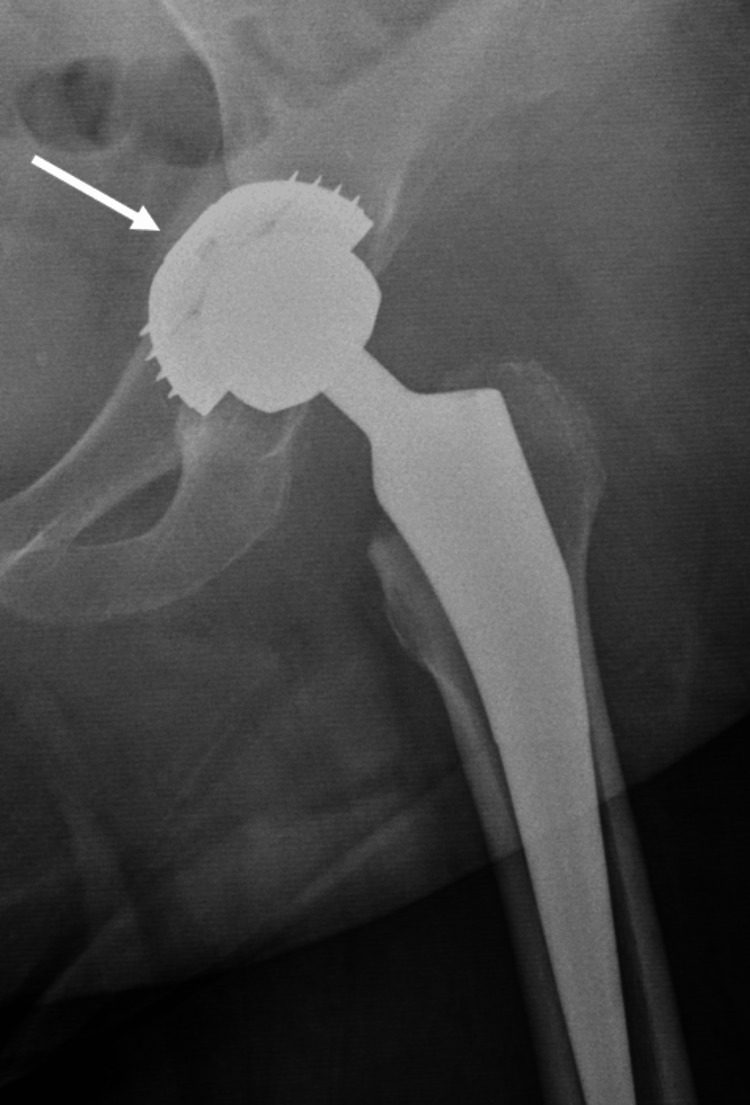
Anteroposterior X-ray view revealing an acetabulum roof fracture (white arrow) that occurred during the placement of the threaded cup

The major complications related to the use of the EcoFit® SC acetabular component are fractures of the acetabulum (e.g., roof, posterior wall) and the incomplete placement of the cup (“very” superficially). We believe that both these complications could be avoided with proper technique and adequate training. Figure 1A exhibits a posterior wall acetabulum fracture, which was observed on the first postoperative day when the patient was mobilized. Open reduction and internal fixation (ORIF) and revision of the cup were performed, as shown in Figure 1B. Moreover, Figure 2 shows an acetabulum roof fracture that occurred intraoperatively.

**Figure 3:**
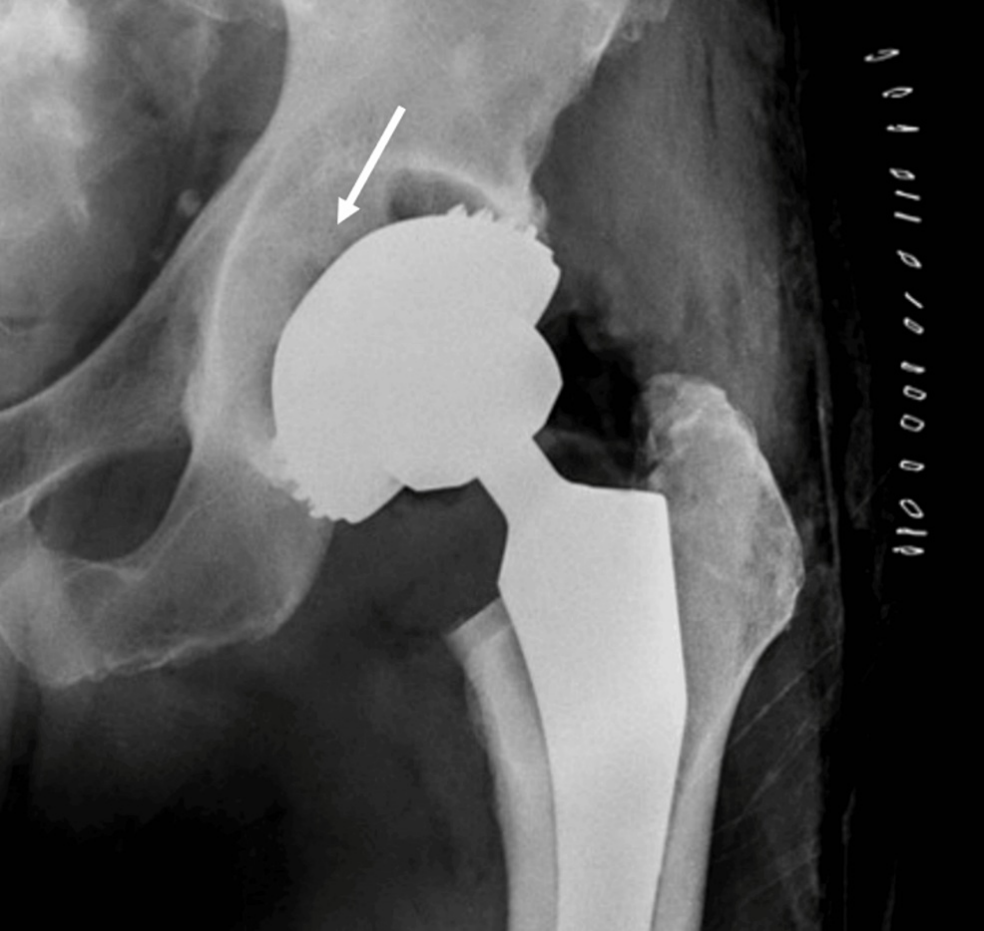
Anteroposterior X-ray view of an incomplete threaded cup placement The white arrow shows the space between the cup and the acetabulum roof. The threads have fixated at the osteophytes of the peripheral rim

Additionally, Figure 3 shows a cup that was firmly fixed at the osteophytes and was placed superficially (incomplete placement), increasing the femoral offset leading to symptomatology similar to trochanteric bursitis. 

**Figure 4:**
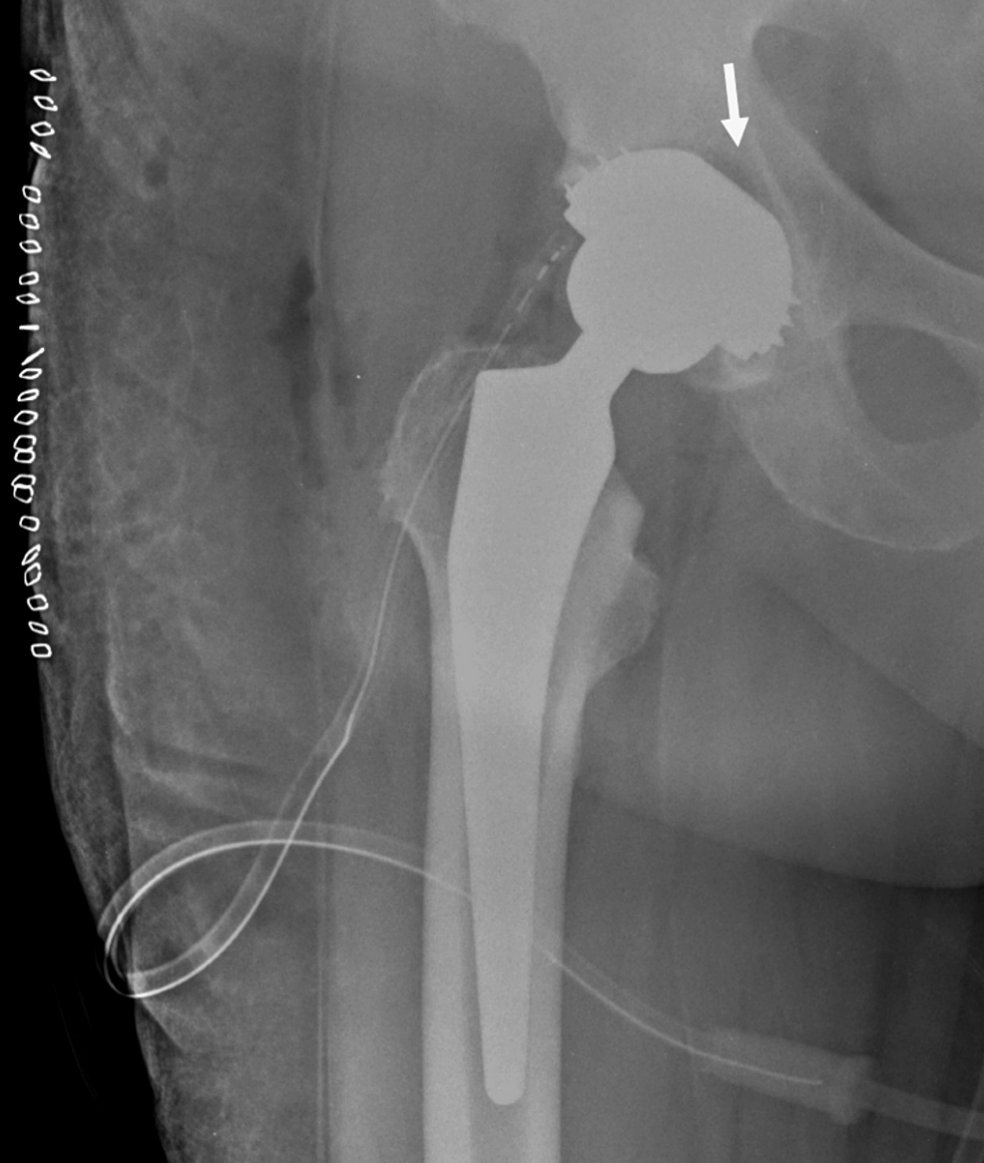
Anteroposterior postoperative X-ray view with the use of the EcoFit® SC threaded cup The white arrow shows the small distance between the acetabulum component and the bone bed

By placing the EcoFit® SC cup in THA, there was a subjective feeling that the component was not placed “deep enough”, leaving a 1-5-mm gap between the component and the acetabular roof, which could also be seen in the postoperative X-ray (Figure 4).

Although such a small distance does not seem to play a crucial role in patient outcomes and implant survival, extreme cases such as the one shown in Figure 3 represent major complications that lead to patient dissatisfaction and the need for revision.

Surgical technique

**Figure 5:**
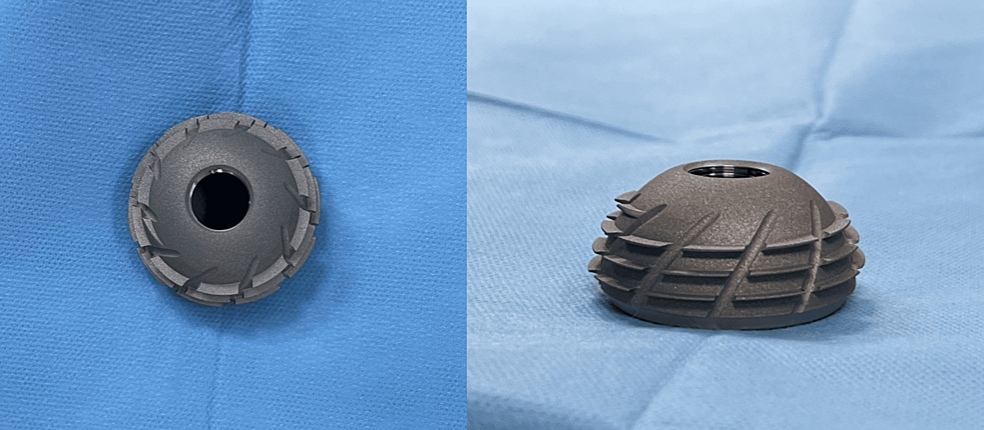
The EcoFit® SC threaded cup

The EcoFit® SC is a third-generation spherical screw-in cup (Figure 5).

To achieve optimal outcomes, preoperative planning and diligent adherence to the surgical plan and the technique are required. Digital, as well as radiographic, templates are available for the EcoFit® SC threaded cup.

**Figure 6:**
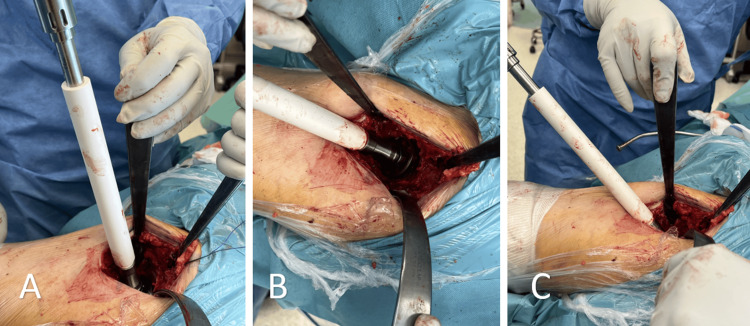
The preparation of the acetabulum with reamers A: Initial vertical position of the small reamer, defining the required depth. B, C: Following the initial vertical reaming, the next ones align anatomically at about 40-45 degrees inclination and 15-20 degrees anteversion

After acetabulum exposure and resection of the articular capsule and labrum, the preparation with external reamers should be initiated. We use the first small external diameter reamer (about 42 mm in the majority of cases that require a 48-52-mm acetabulum component according to preoperative planning) in a vertical manner. This way, we define the required depth. After reaching the required depth with the first reamer, the next one, e.g., 44 mm, is aligned anatomically, at 40-45 degrees inclination and 15-20 degrees anteversion in most cases. Figure 6 illustrates the preparation of the acetabulum with reamers.

It should be noted that in most cases, we utilize the modified anterolateral minimal invasive hip surgery technique (ALMIS) [9,10]; hence, in posterior-approach cases, the anteversion should be greater, e.g., 20-30 degrees.

To avoid major complications with the EcoFit® SC threaded cup, it is critical to preserve adequate bone bed at the posterior and anterior walls. This has to be manually checked intraoperatively after the use of each reamer. If the surgeon feels that the walls have become too thin, the placement of a threaded component should be avoided. It should be kept in mind that torque forces during insertion of a threaded cup are much higher than when inserting a press-fit one. Hence, threaded cups should not be utilized in cases where the acetabulum lacks sufficient thickness to accommodate proper reaming without the risk of significant floor perforation, such as the one shown in Figure 2. Additionally, caution should be exercised when enlarging the acetabular size, as this may lead to the walls becoming too fragile to adequately support a threaded cup, as occurred in the case shown in Figure 1. The thin remaining posterior wall could not withhold the torque forces during the cup’s placement, leading to a fracture later during weight-bearing. Placement of a press-fit cup or a cemented one may prove better in these cases, which helps avoid these major intraoperative complications.

Another major issue encountered in the placement of the EcoFit® SC threaded acetabulum component involves the preparation of the peripheral rim, especially superficially in the "entrance" of the acetabulum. After reaching the depth with the first small external diameter reamer, the surgeon should focus on enlarging the peripheral rim in such a way that the threads of the cup will not lock superficially thereby leading to incomplete component placement. Hence, the reamer should be used in an "inwards-outwards" manner to facilitate adequate room for the insertion of the cup, as shown in Video 1.

**Video 1:** Preparation of the rim of the acetabulum for the insertion of a threaded cup

Progressively larger reamers are used until the reamer is enclosed completely within the acetabulum. The acetabulum is prepared until the bleeding subchondral bone is reached. The size may also be confirmed by using the trial shells, although the trial shells should not be necessarily fixed firmly in threaded components, since the initial stabilization is provided through the threads. The screwing-in instrument is assembled and the cup is screwed in, manually at first, and clockwise, until a minor level of stability is achieved, and then with the extension sleeve and the chest plate. At this stage, the chest plate provides stable inclination and anteversion. The acetabular cup should be screwed in deep until the resistance is increased considerably and the cup reaches the base of the acetabulum. If necessary, the insertion instrument should be unlocked and it should be checked whether the cup is already in contact with the base of the acetabulum.

## Discussion

The earlier generations of threaded cup designs featured a smooth surface, as it was initially believed that relying solely on mechanical interlocking between the bone and the metal shell would offer satisfactory initial fixation and long-term stability [4,11]. However, it became apparent that depending solely on primary fixation was insufficient to guarantee the implant's long-term durability. The modern acetabular cup designs feature a metallic hemispheric shell with a rough surface to enhance osseous integration, thereby ensuring long-term durability [6,11]. Third-generation cups also incorporate modular metal, ceramic, and modified polyethylene inserts. Both second and third-generation threaded cups have demonstrated promising outcomes in early and intermediate follow-up periods [6,11,12].

Each material possesses certain specific characteristics that the surgeon should be familiar with, in order to utilize it optimally for the benefit of the patients. The EcoFit® SC is a third-generation spherical screw-in cup. It is an acetabular component intended to be used in combination with an acetabular cup insert to replace the natural acetabulum in THA. It is intended for cementless, screw-in fixation. It has 3.5 circles of threads providing initial stability, as well as a porous surface suitable for secondary biological integration [13]. With the use of the EcoFit® SC threaded cup, there was a subjective feeling that the component was not placed “deep enough”, leaving a 1-5-mm gap between the component and the acetabular roof, as shown in Figure 4. This has been documented in another study as well [14]. Thorwächter et al. suggested that the extension of the acetabulum cavity to 51 mm while using the Implantcast EcoFit size 50 should be implemented in clinical applications [14]. They achieved sufficient rim preparation by over-reaming by 1 mm.

We presented an alternative way that involves an "inwards-outwards" preparation of the acetabulum rim with the same-size reamer (e.g., 50-mm reamer for 50-mm components), ensuring that the cup will be placed properly and not get anchored superficially, as shown in Video 1. The incomplete placement could be attributed to the fact that the threaded acetabulum components have a larger diameter than that of the preparation reamer and the threads may be anchored superficially at the rim or the osteophytes of the acetabulum. These implants provide excellent initial stability and, hence, after this fixed placement, it is extremely difficult to change the positioning of the component. Nevertheless, it should be noted that implant survival and patient outcomes have been recorded to be excellent. We observed non-infection-related implant survival of up to 99.7% (0.3% revision due to aseptic loosening) and a median Harris hip score of 94 in patients at 6.9 years of follow-up [8].

There are some cases where the threaded cups are contraindicated. Firstly, there are contraindications related to the specific anatomy of the patient’s acetabulum. In dysplastic hips, such as type A dysplasia according to the Hartofilakidis classification, where there is a segmental deficiency of the superior wall and inadequate depth of the true acetabulum, a threaded cup is probably not the best option, since there is insufficient bone bed for the anchoring of the threads [15]. Moreover, in cases where the acetabulum walls are insufficient (more than one-third of the acetabular ring) and bone grafts are required, it is advisable to refrain from using a threaded cup and instead consider the use of a bipolar cup. These cases should be recognized during the preoperative templating and planning. Secondly, some contraindications may arise intraoperatively. In cases where the acetabulum becomes too thin during reaming, the risk of significant floor perforation increases, and threaded cups should also be avoided. Additionally, if the acetabulum walls become too thin during preparation, the placement (screwing-in) of a threaded cup highly increases the torque forces, which could lead to intraoperative fractures, and hence a threaded cup cannot be supported.

## Conclusions

Each acetabulum component has certain specific characteristics that the surgeon should be familiar with, in order to optimize implantation and clinical results. The EcoFit® SC threaded cup has displayed excellent clinical results in the past; however, some major complications have also been observed. It is important to thoroughly prepare the peripheral rim of the acetabulum to avoid incomplete placement; also, in cases where the posterior or anterior walls have become too thin, the insertion of a threaded cup should be avoided to prevent an intraoperative fracture due to the torque forces applied at the acetabulum during placement.
